# Smoking Ban Policies in Italy and the Potential Impact of the So-Called Sirchia Law: State of the Art after Eight Years

**DOI:** 10.1155/2014/293219

**Published:** 2014-05-15

**Authors:** Maria Rosaria Gualano, Fabrizio Bert, Giacomo Scaioli, Stefano Passi, Giuseppe La Torre, Roberta Siliquini

**Affiliations:** ^1^Department of Public Health, University of Turin, via Santena 5 bis, 10126 Turin, Italy; ^2^Department of Public Health and Infectious Diseases, Sapienza University of Rome, Piazzale Aldo Moro 5, 00185 Rome, Italy

## Abstract

*Objective*. The aim of the present work is to describe the state of the art of tobacco habits in Italy, eight years after the law was introduced. *Methods*. Time series analyses, based on estimates of smoking prevalence/consumption derived from the openly available data of national surveys performed during the 2001–2013 period, were performed. Data have been expressed in percentage of smokers and daily cigarettes consumption. Time changes are expressed as expected annual percentage change (EAPC). *Results*. Over time, the percentage of Italian smokers shows a constant and statistically significant decrease (from 28.9% in 2001 to 20.6% in 2013, EAPC = −2.6%, and *P* < 0.001). Regarding data stratified by gender, we found a stronger reduction among men (EAPC = −2.9%, *P* < 0.001) than in women (EAPC = −2.5%, *P* < 0.001). Similarly, the consumption of tobacco smoking, measured as the number of daily cigarettes smoked, registered a downward trend (*P* < 0.001). No join point (time point when a significant trend change is detected) resulted from the trend analysis. *Conclusions*. Data show a constant decrease of tobacco consumption in Italy, with no join point related to the introduction of the banning law. These findings require to reflect on the priorities of the smoking banning policies that may be focused on other intervention activities such as to increase the price of cigarettes.

## 1. Introduction

Currently, smokers in Italy are around 11 million: of them, 43.5% are women [1]. In Italy tobacco use is an important public health issue, being the first preventable cause of death (the third one is passive smoking) [2].

Lung cancer kills 36,000 Italians each year [3] and smoking-related deaths are nearly 72,000, accounting for 12.5% of total deaths in Italy [4].

Over 25% of smoking-related deaths occur among individuals aged between 35 and 65 [5]. In addition, Russo and Scafato calculated that around 15% of total hospitalizations can be related to smoking effects, with an economic cost to the health system accounting for 3 billion euros (6.7% of national health costs) [5].

Since 1975 in Italy several laws aimed at controlling tobacco use have been enacted. In particular, from 10 January 2005, the law number 3 of 16 January 2003 (the so-called Sirchia Law named after the Health Ministry who promoted it) that banned smoking in all indoor public places was into force. This represents one of the first smoke-free legislation introduced in Europe, aiming at controlling smoking habits in all the public and private places such as bars and restaurants in order to protect nonsmokers. Afterwards, Italy, with the law number 75 of 18 March 2006, has ratified the Framework Convention of the World Health Organization to control and fight tobacco smoking. This convention can be considered a milestone for the promotion of public health and provides new horizons for international health cooperation. Such global initiatives have achieved important goals: to date, about 2.3 billion people are now covered by at least one tobacco control measure at the highest level of achievement [6]. Interestingly, a suitable scale was created by an international experts panel who proposed the Tobacco Control Scale (TCS) in order to evaluate the quality of implementation of tobacco control policies across European countries. In particular, the scale considers six specific policies to be implemented: bans and restrictions on smoking in public places and workplaces, cigarette taxation, public information campaigns, bans on the advertising and promotion of tobacco products, health warnings on tobacco product packaging, and treatment to help quitting [7].

In this framework, there are still few research experiences investigating the impact of the introduction of the smoking banning policies worldwide: for instance, in Germany, Anger et al. found that the introduction of smoking bans in 2007-2008 did not change smoking behaviour in the whole population, but only selected groups (men and young and unmarried people, as well as for those living in urban areas) were positively influenced by the law [8]. A recent study carried out in the USA did not find evidence that smoking bans, either in workplaces or in bars and restaurants, have a real effect on smoking behavior, in terms of consumption and smoking cessation [9].

An Italian study published in 2013 investigated only the short-term effect of the Sirchia law, by analyzing differences in smoking behavior between 2004 and 2005. They found that, immediately after the introduction of such a law, Italian smokers changed their habits [10]. Moreover, a previous Italian study conducted before and after the introduction of the indoor smoking ban (2001–2006) reported that the introduction of the ban improved the efficacy of smoking cessation treatments at 1-year follow-up [11].

The present work aims to describe the state of the art of tobacco habits in Italy, eight years after the banning law was introduced in 2005, by using a time trend analysis in order to show the long-term effects of such a law.

## 2. Methods

The openly available online data of tobacco consumption of national surveys commissioned by the National Institute of Health in collaboration with the Mario Negri Institute for Pharmacological Research and the Italian Cancer League (LILT) and conducted annually by the DOXA Institute of Statistics (the Italian branch of the Gallup International Association) have been elaborated. The DOXA Institute interviews a representative sample comprising more than 3,000 Italian citizens aged more than 15 regarding the prevalence, the attitudes, and the behaviors of Italian smokers each year [1]. The time period considered was 2001–2013.

## 3. Statistical Analysis

Data have been expressed in percentage of smokers and daily cigarettes consumption. Furthermore, data were stratified by age (age groups: 15–24, 25–44, 45–64, and 65+) and gender, where available. Authors were not able to retrieve data stratified by age for the year 2001, so these analyses were performed for the period from 2002 to 2013. In order to obtain the time trends of tobacco consumption, the following formula was applied for logarithmic transformation of the consumption rates:

(1) $$\begin{array}{c} \ln\left(\text{rate}\right)=b\times \text{years}, \end{array}$$

where “*x*” represents the calendar years, “*b*” is the regression coefficient, and “*y*” is the incidence rate.

In particular, a join point represents the time point when a significant trend change is detected. Time changes are expressed as expected annual percentage change (EAPC) with the respective 95% confidence interval (95% CI); significance levels of time trends are also reported. The null hypothesis was tested using a maximum of 3 changes in slope with an overall significance level of 0.05 divided by the number of join points in the final model. Linear graphs were created to represent trends. Statistical analysis was conducted by using the join point regression program software version 4.0. The Poisson model was applied to control heteroskedasticity in the population [12, 13].

## 4. Results

**Figure 1:**
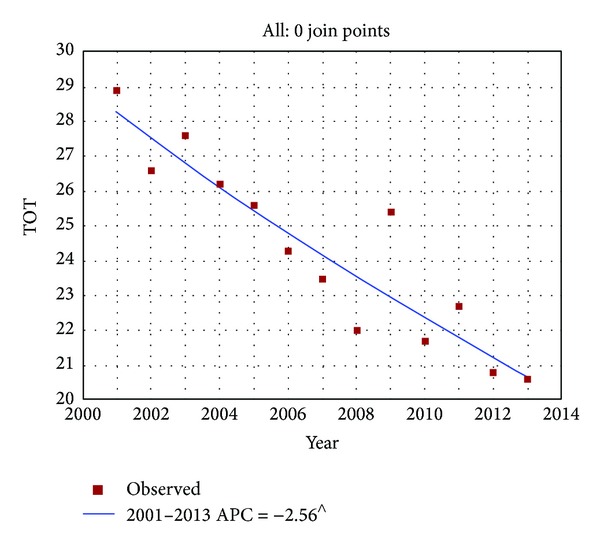
Time trends of annual percentage of tobacco smokers in Italy (2001–2013). ^∧^Statistically significant results APC = annual percentage change.

Over time, the percentage of Italian smokers shows a constant and statistically significant decrease (from 28.9% in 2001 to 20.6% in 2013, EAPC = −2.6%, and *P* < 0.001); see Figure 1. Regarding data stratified by gender, we found a stronger reduction among men (EAPC = −2.9%, *P* < 0.001) than in women (EAPC = −2.5%, *P* < 0.001).

**Figure 2:**
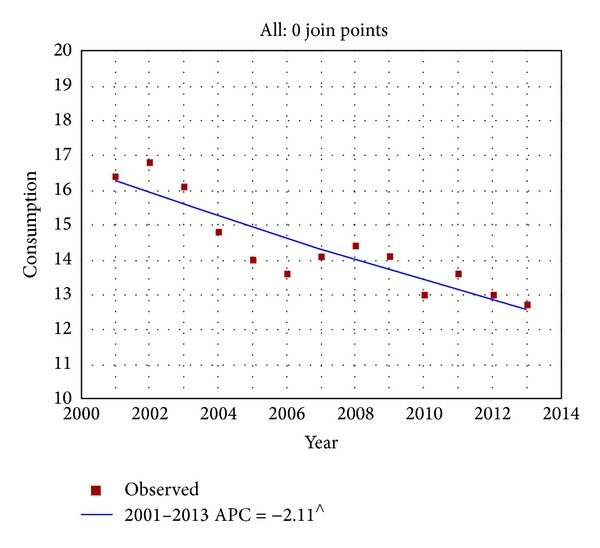
Time trends of tobacco consumption (daily cigarettes smoked) smokers in Italy (2001–2013). ^∧^Statistically significant results APC = annual percentage change.

Similarly, the consumption of tobacco smoking, measured as the number of daily cigarettes smoked, registered a downward trend (from 16.4 cig/day in 2001 to 12.7 cig/day in 2013, EAPC = −2.1%, and *P* < 0.001); see Figure 2. Interestingly, Italian women seem to have lesser propensity to reduce the number of daily cigarettes than men. Indeed, in 2001, women used to smoke 12.2 cig/day and in 2013 11.5 cig/day (EAPC = −1, *P* = 0.03) while men have reduced their consumption from 18.8 cig/day to 13.5 cig/day in the same time period (EAPC = −2.5, *P* < 0.001).

Data on the prevalence of smokers and tobacco consumption (overall data and data stratified by gender) are shown in Table 1.

No statistically significant join point resulted from the trend analysis. Nevertheless we have to acknowledge that in 2009 an increase in smoking prevalence was registered (from 22% in the previous year to 25.4% in 2009). In 2010 the prevalence registered a decrease (21.7%). Notwithstanding, this trend change was not statistically significant.

Regarding data stratified by age groups, we retrieved that women belonging to extreme age groups were more likely to stop smoking than men and women of the other groups: indeed women older than 65 years and aged 15–24 yielded EAPC values of −4.18% (*P* = 0.02) and −3.86% (*P* = 0.02), respectively (in the time period 2002–2013). Particularly, women aged 45–64 seem to increase their tobacco consumption, showing the only positive value of EAPC = +0.90; nevertheless this finding does not result in being statistically significant (*P* = 0.40). All the results of the trend analysis stratified by age group are showed in Table 2.

## 5. Discussion and Conclusions

The present work analyzes the possible long-term impact of the so-called Sirchia Law that since 2005 banned smoking in all public places in Italy. Our data show a statistically significant decrease of tobacco consumption, constantly lasting from 2001 and registering no join point related to the introduction of the banning law.

A previous Italian study by Federico et al. aimed at evaluating the immediate as well as the longer-term impact of the Sirchia law on smoking in the overall population and by educational level in the period until 2010 and concluded that the Italian smoke-free policy on smoking seems to be for short term only. Indeed, banning policies may not achieve the secondary effect of reducing smoking prevalence in the long term, and they may have limited effects on inequalities in smoking [14].

Based on the results of the present study and inline with previous data, we can affirm that since 1990 in Italy there was a downward trend of tobacco consumption [1]. Even though Italian women used to smoke less than men (4.7 million versus 6.1 million), we found a lower percentage of reduction among women (EAPC = −2.5%, *P* < 0.001) than in men (EAPC = −2.9%, *P* < 0.001). Probably this can be related to the female propensity to smoke in order to control negative mood/affect, depression, and/or postcessation weight gain [15].

Nevertheless, by analyzing the stratified data, it arose that women older than 65 years and aged 15–24 are more likely to quit smoking than men and women of the other age groups. These differences by gender, particularly among adolescents, in this kind of behavior are reported in literature also [16]. In addition, a study published in 2006 demonstrated that older women are more likely to quit smoking [17].

An interesting fact was registered in 2009, when a peak of increasing prevalence was registered, mainly attributable to former smokers in 2008 who relapsed [18]. Considering that the 2009 survey was carried out during the period of the peak recession in Italy, a possible explanation for this unexpected data, as reported by the current literature, could be the economic crisis that, by causing psychosocial stress, increases tobacco consumption [19–21].

Regarding the consequences of this banning law, current literature reports that the bans were largely respected, with positive effects in particular on passive smoking, and it appears that in Italy the smoke-free law did not affect the business of restaurants and bars [22].

Moreover, a possible synergistic effect of banning policies and smoking cessation methods (such as bupropion) seems to exist [11].

In addition, other studies have suggested that smoking bans should decrease the phenomenon of the “social acceptability” of smoking, registering a reduction of smoking habits in private places and houses also. For instance, in Italy in 2006 more than 50% of people interviewed declared that their guests could smoke only outside of their home [23].

Given this context, we suggest to reflect on the priorities of the smoking banning policies that may be focused on other intervention activities to discourage tobacco habits, such as to increase the price of cigarettes, to cover the pharmacological supports for smoking cessation, and to introduce health warnings on cigarette packages. Concerning the last measure, the European Union on 7 March 2012 adopted 14 new health warnings to appear on cigarettes' packs. In this regard, a recent Italian study showed some positive effects of the introduction of health warnings on cigarette packages. In fact, almost all the smokers interviewed were informed on tobacco effects, 14% of them reduced the amount of daily smoking, and 5% attempted to quit [24].

Additionally, the role of the health professionals in helping their patients/citizens to quit smoking has to be considered [25, 26]. Given the high prevalence of smokers among these kinds of professionals [27, 28], it would be important to develop some specific actions of promoting the smoking cessation addressed to the health workers, in order to create a cascade effect, from the healthcare professionals to the patients/citizens.

Interestingly, about the future trends, international projections to 2025 that smoking prevalence and smoking-attributed mortality will decrease in parallel in most developed countries towards lower limits that are not yet defined [29].

The present work is affected by some limitations that should be acknowledged. First of all, our trends' calculations are based on aggregate data, not considering the individual level. In consequence of this weakness, our findings should be interpreted considering the limitations that affect the ecological studies. Moreover, data are based on surveys registering self-reported data, thus giving a possible underestimation of the smoking prevalence. The other limits are related to the possible biases occurring in the population-based surveys, such as information and recall bias. Nevertheless, the highly representative nature of the sample makes these data very accurately estimated [22].

## 6. Conclusions

Given that Italy has now reached the final stage of the tobacco epidemic, antismoking strategies should focus on support for smoking cessation. Given our results, Italy should introduce new policies and measures for tobacco control. For instance, to increase tobacco taxes and prices and to introduce the coverage for smoking cessation treatment in the national health system are highly recommended. Furthermore, since Italy was one of the first countries to introduce such a law in Europe, it will be interesting to analyze data from all the other European countries, when available, in order to assess the global impact of the smoking banning laws in Europe. The future priorities of the public health agenda may be focused on promoting other intervention activities to discourage tobacco habits among the European and Italian citizens.

## Figures and Tables

**Table 1:** Percentage of smokers and daily cigarettes consumption (overall data plus data stratified by gender). Italy, 2001–2013.

Calendar year	Percentage of smokers (overall)	Percentage of smokers (males)	Percentage of smokers (females)	Daily cigarettes consumption (overall)	Daily cigarettes consumption (males)	Daily cigarettes consumption (females)
2001	28.9	34.8	23.6	16.4	18.8	12.2
2002	26.6	31.1	22.3	16.8	19.2	13.1
2003	27.6	33.2	22.5	16.1	18.6	12.2
2004	26.2	30.0	22.5	14.8	16.1	13.1
2005	25.6	29.3	22.1	14.0	15.8	11.9
2006	24.3	28.6	20.3	13.6	14.8	12.0
2007	23.5	27.9	19.3	14.1	14.9	13.0
2008	22.0	26.4	17.9	14.4	15.3	13.0
2009	25.4	28.9	22.3	14.1	16.0	11.7
2010	21.7	23.9	19.7	13.0	14.5	11.5
2011	22.7	26.0	19.6	13.6	15.6	11.1
2012	20.8	24.6	17.2	13.0	14.3	11.3
2013	20.6	26.2	15.3	12.7	13.5	11.5

**Table 2:** Expected annual percentage change (EAPC) and 95% confidence interval (CI) of tobacco consumption (data stratified by age and gender).

Expected APC^a^ of tobacco consumption (2002–2013)				
Age group	EAPC^a^	95% CI	*P* value	
15–24	−3.45	(−5.7; −1.2)	0.02	Males
25–44	−1.98	(−3.3; 0.7)	0.01	
45–64	−1.16	(−2.8; +0.2)	0.07	
65+	−1.94	(−5.7; +2.0)	0.20	

15–24	−3.86	(−6.5;−0.9)	0.02	Females
25–44	−3.56	(−5.3; −1.9)	0.002	
45–64	+0.90	(−1.5; 2.9)	0.40	
65+	−4.18	(−6.8; −1.5)	0.02	

^a^APC: annual percentage change.
